# On the Use of the Bark and Leaves of the Olive-tree

**Published:** 1846-12

**Authors:** 


*On the Use of the Bark and Leaves of the Olive-tree.*—M. Spinelli administers the bark and leaves of the olive-tree in the following forms: extract, tincture, decoction, syrup, and powder. The extract of the bark is intensely bitter, and that prepared from the leaves possesses a very agreeable aroma. M. Spinelli has found preparations from the olive-tree of much use in atonic affections of the *primæ viæ*, and of the mucous membranes in general, and in chronic diarrhœa, and dysentery. When combined with preparations of iron, M. Spinelli has administered it with advantage in leucorrhœa and chlorosis. *Ibid.*

